# Relationship between antihypertensive drug classes and ambulatory blood pressure profile in patients with chronic kidney disease

**DOI:** 10.1038/s41371-026-01216-8

**Published:** 2026-09-02

**Authors:** Fotini Iatridi, Marieta Theodorakopoulou, Artemios G. Karagiannidis, Pantelis Sarafidis

**Affiliations:** First Department of Nephrology, Aristotle University of Thessaloniki, Hippokration Hospital, Thessaloniki, Greece

**Keywords:** Hypertension, Diagnosis

Ambulatory blood pressure monitoring (ABPM) represents the gold standard for blood pressure (BP) measurement and offers important advantages by revealing abnormal BP profiles, such as white coat, masked uncontrolled, and nocturnal hypertension, which cannot be captured by office BP measurements alone [1, 2]. Among the various ambulatory BP phenotypes, nocturnal hypertension has emerged as one of the strongest predictors of cardiorenal risk and is particularly common in patients with chronic kidney disease (CKD) [3, 4]. Yet despite growing evidence supporting its clinical relevance, nighttime BP remains largely overlooked in routine practice, where therapeutic decisions continue to be driven predominantly by office BP readings. In this issue, Pasricha et al. report an analysis from the Chronic Renal Insufficiency Cohort (CRIC) examining the relationship between antihypertensive medication classes and ambulatory BP profiles in patients with CKD [5].

The study included nearly 1500 individuals with CKD who underwent ABPM and evaluated associations between major antihypertensive drug classes and several BP phenotypes, including masked uncontrolled hypertension, nocturnal hypertension, non-dipping status, and BP variability [5]. The authors observed that renin–angiotensin system (RAS) blockers were associated with lower odds of sustained hypertension and nocturnal hypertension, whereas β-blockers were associated with higher odds of masked uncontrolled hypertension and nocturnal hypertension. Calcium channel blockers (CCBs) were also associated with nocturnal hypertension and non-dipping status, although they appeared to reduce short-term BP variability [5].

Although cross-sectional and hypothesis-generating, these findings are clinically relevant because they focus on evaluation of several high-risk ambulatory BP phenotypes which are strongly associated with adverse outcomes in CKD patients and provide an opportunity to reconsider antihypertensive therapy through the lens of ambulatory BP. One of the most important observations was a clear association between RAS blockers use and lower odds of both sustained hypertension and nocturnal hypertension [5]. These findings are biologically plausible and clinically relevant. In CKD, activation of the RAS contributes not only to elevated BP but also to sodium retention, vascular dysfunction, and abnormal circadian BP rhythm [6]. The association of RAS blockade with a more favorable ambulatory BP profile across both daytime and nighttime domains suggests that improved 24 h BP control may be a key mechanism contributing to the cardiorenal benefits of these agents [6]. Conversely, these findings also suggest that studies relying solely on office BP may underestimate the extent of BP-related benefit achieved with RAS blockade. At the same time, residual confounding cannot be excluded, given that RAS blockers are recommended and prescribed as first-line therapy in patients with albuminuric CKD [1].

The association between β-blocker use and masked uncontrolled hypertension is also interesting. Masked hypertension represents one of the most clinically challenging phenotypes because patients appear adequately controlled during office visits while remaining exposed to elevated BP and increased cardiovascular risk [1]. In the present study, nearly one-third of participants exhibited masked hypertension despite ongoing treatment and β-blocker therapy was associated with higher odds of this phenotype [5]. Based on these observations there is a possibility that some antihypertensive regimens may disproportionately influence office and ambulatory BP measurements. B-blockers may lower office BP more effectively than ambulatory BP by blunting sympathetic responses to the clinical encounter, thereby reducing stress-related elevations that contribute to office measurements. At the same time, many patients with CKD and hypertension have volume expansion, arterial stiffness, and altered sodium handling–pathophysiological processes that may be less responsive to β-adrenergic blockade and continue to sustain elevated BP throughout daily activities and during the nighttime period [3]. In addition, some β-blockers may provide less consistent 24 h BP control than other antihypertensive classes, particularly when dosing schedules or pharmacokinetic profiles leave periods of incomplete coverage. Although residual confounding and treatment selection bias remain plausible explanations, these mechanisms could contribute to a greater discrepancy between office and amulatory BP. Interestingly, these observations also align with the fact that most hypertension guidelines recommend RAS blockers, in combination with CCBs or thiazide/thiazide-like diuretics, as preferred initial therapy for most patients, whereas β-blockers are generally reserved for individuals with specific cardiovascular indications. Although the present cross-sectional analysis cannot establish causality or compare treatment efficacy, the observed associations provide some indirect support for this treatment approach, highlight the importance of out-of-office BP measurements and strengthen the rationale for wider implementation of ABPM in CKD populations, particularly when treatment decisions are guided solely by office BP readings.

More than half of the cohort exhibited nocturnal hypertension and a similar proportion demonstrated a non-dipping pattern [5]. These observations are consistent with findings from other cohorts [7] and underscore the remarkable burden of abnormal nighttime BP profiles in CKD [3]. Numerous studies have demonstrated that nighttime BP is more strongly associated with cardiovascular events, kidney disease progression, and mortality than office BP and, in many settings, even daytime ambulatory BP [3, 7, 8]. The strong prognostic value of nocturnal BP likely reflects the fact that it captures fundamental disturbances in volume status, sympathetic activation, vascular stiffness, sleep-related disorders, and circadian rhythm that are particularly prevalent in CKD [3]. Against this background, the associations observed with specific antihypertensive classes become particularly striking. The lower odds of nocturnal hypertension among RAS blockers users are consistent with the central role of the RAS in both hypertension and circadian BP regulation. In contrast, β-blockers and CCBs were associated with higher odds of nocturnal hypertension, while CCBs were additionally associated with non-dipping [5]. Although these observations should not be interpreted as evidence of harm, they raise important mechanistic questions regarding how different antihypertensive therapies interact with the pathophysiology of nighttime BP regulation.

In addition, although CCBs were associated with greater odds of nocturnal hypertension and non-dipping, they were simultaneously associated with lower short-term BP variability [5]. This finding is consistent with prior small interventional evidence in diabetic hypertensive patients, in whom the dihydropyridine CCB lacidipine reduced both 24 h BP and 24 h BP variability, with concomitant improvement in baroreflex sensitivity [9]. This apparent dissociation illustrates the complexity of ambulatory BP physiology: BP variability, nocturnal dipping, and mean ambulatory BP levels are related but reflect distinct biological processes, and improvement in one domain does not necessarily translate into improvement in another, as we have also previously observed in randomized studies across different hypertensive and CKD populations [10, 11].

Importantly, the study [5] also highlights the limitations of our current therapeutic framework. Antihypertensive medications are generally selected according to office BP levels, comorbidities, and guideline-based treatment algorithms [1], whereas ambulatory BP phenotypes rarely influence treatment decisions. Yet the present findings suggest that specific ambulatory BP profiles may be associated with particular therapeutic regimens. Whether this reflects causal drug effects or treatment selection cannot be determined from a cross-sectional analysis. Several other important limitations influence interpretation of these findings. Most importantly, the CRIC dataset did not include granular information on antihypertensive medication dose, timing of administration, duration of action (short- vs long-acting agents), or treatment adherence [5]. These considerations are particularly relevant because ambulatory BP phenotypes are inherently time-dependent; effect of an antihypertensive agent on nocturnal BP may depend not only on its pharmacological class but also on whether adequate therapeutic coverage extends throughout the night. Likewise, the inability to distinguish among individual agents within each class limits mechanistic interpretation, as substantial heterogeneity exists even among drugs sharing the same broad pharmacological category.

These limitations are particularly relevant in the context of ongoing discussions regarding chronotherapy. In the general hypertensive population, chronotherapy remains controversial. Although meta-analyses suggest that bedtime dosing may reduce nighttime BP [12], methodological concerns persist, and large pragmatic trials have not demonstrated a clear cardiovascular benefit of evening over morning administration [13, 14]. Accordingly, current recommendations emphasize the use of long-acting agents that provide consistent 24 h BP control [1, 3] rather than routine bedtime dosing for all patients. Evidence in CKD remains limited; to date, the only study conducted in non-dialysis CKD patients showed that shifting one antihypertensive drug from morning to evening dosing decreased nocturnal BP and restored dipping status to normal [15]. Future studies integrating drug class, dosing schedule, duration of action, and nocturnal BP assessment are needed to determine whether specific ambulatory BP phenotypes are more responsive to therapeutic timing, drug selection, or adequate 24 h pharmacological coverage.

Overall, the most important contribution of this study [5] may be that it highlights the inadequacy of relying on office BP measurements alone in patients with CKD. Broader implementation of ABPM should be prioritized to detect masked uncontrolled hypertension, nocturnal hypertension, non-dipping, and other ambulatory BP phenotypes that remain undetectable from the routine office assessment. As ABPM becomes more integrated into routine clinical practice, treatment paradigms may need to focus not only on achieving target office BP, but also on identifying and treating specific ambulatory BP phenotypes that drive cardiovascular and renal risk in patients with CKD. The present study indirectly reinforces this concept. If different antihypertensive classes exhibit differential benefits with abnormal BP phenotypes, then future investigations must move beyond simple drug-class comparisons and incorporate detailed assessments of treatment timing and duration of action. Understanding whether nocturnal hypertension is best addressed by particular drug classes, optimizing dosing schedules, choosing longer-acting formulations, or combining these approaches represent important questions to be answered. Until then, clinicians should recognize ambulatory BP as a critical component of risk assessment and consider wider use of ABPM in patients with CKD, particularly when office BP appears controlled despite ongoing evidence of cardiovascular or renal risk.
